# Transcriptome Profile Reveals Drought-Induced Genes Preferentially Expressed in Response to Water Deficit in Cultivated Peanut (*Arachis hypogaea* L.)

**DOI:** 10.3389/fpls.2021.645291

**Published:** 2021-04-30

**Authors:** Xu Wang, Xinlei Yang, Yucheng Feng, Phat Dang, Wenwen Wang, Rita Graze, Josh P. Clevenger, Ye Chu, Peggy Ozias-Akins, Corley Holbrook, Charles Chen

**Affiliations:** ^1^Department of Crop, Soil and Environmental Sciences, Auburn University, Auburn, AL, United States; ^2^State Key Laboratory of North China Crop Improvement and Regulation, Laboratory of Crop Germplasm Resources of Hebei, Hebei Agricultural University, Baoding, China; ^3^United States Department of Agriculture–Agricultural Research Service National Peanut Research Laboratory, Dawson, GA, United States; ^4^Department of Biology, Auburn University, Auburn, AL, United States; ^5^HudsonAlpha Institute for Biotechnology, Huntsville, AL, United States; ^6^Center for Applied Genetic Technologies, University of Georgia, Tifton, GA, United States; ^7^United States Department of Agriculture–Agricultural Research Service Crop Genetics and Breeding Research, Tifton, GA, United States

**Keywords:** transcriptome, drought, induced genes, cultivated peanut, water deficit

## Abstract

Cultivated peanut (*Arachis hypogaea*) is one of the most widely grown food legumes in the world, being valued for its high protein and unsaturated oil contents. Drought stress is one of the major constraints that limit peanut production. This study’s objective was to identify the drought-responsive genes preferentially expressed under drought stress in different peanut genotypes. To accomplish this, four genotypes (drought tolerant: C76-16 and 587; drought susceptible: Tifrunner and 506) subjected to drought stress in a rainout shelter experiment were examined. Transcriptome sequencing analysis identified that all four genotypes shared a total of 2,457 differentially expressed genes (DEGs). A total of 139 enriched gene ontology terms consisting of 86 biological processes and 53 molecular functions, with defense response, reproductive process, and signaling pathways, were significantly enriched in the common DEGs. In addition, 3,576 DEGs were identified only in drought-tolerant lines in which a total of 74 gene ontology terms were identified, including 55 biological processes and 19 molecular functions, mainly related to protein modification process, pollination, and metabolic process. These terms were also found in shared genes in four genotypes, indicating that tolerant lines adjusted more related genes to respond to drought. Forty-three significantly enriched Kyoto Encyclopedia of Genes and Genomes pathways were also identified, and the most enriched pathways were those processes involved in metabolic pathways, biosynthesis of secondary metabolites, plant circadian rhythm, phenylpropanoid biosynthesis, and starch and sucrose metabolism. This research expands our current understanding of the mechanisms that facilitate peanut drought tolerance and shed light on breeding advanced peanut lines to combat drought stress.

## Introduction

Cultivated peanut (*Arachis hypogaea* L.) is an important legume that is grown mainly on arid and semiarid regions where peanut productivity is usually limited by water deficit (Pimratch et al., 2008, 2009; Songsri et al., 2008; Balota et al., 2012; Dinh et al., 2013; Brasileiro et al., 2015). Drought stress during the mid-growing season from flowering to pod development leads to a severe reduction in peanut pod yield due to the highest water requirement during this period (Stansell and Pallas, 1985; Sterling et al., 1989). How to sustain and even increase peanut production to meet growing population needs, whereas environmental conditions are deteriorating, is a major challenge that the peanut industry faces. Developing drought-tolerant varieties adapted to various levels of drought stress is a priority for many peanut breeding programs (Zhao et al., 2018). Unfortunately, traditional breeding approaches achieve little progress because drought-stress tolerance is a polygenic trait, and little is known about the molecular signaling and regulatory mechanisms of peanuts under drought stress.

The vulnerability of peanut to drought stress depends on genotypic variability (Stansell and Pallas, 1985; Greenberg et al., 1992; Puangbut et al., 2009; Devi et al., 2010; Dinh et al., 2013). Genotypic variations in several physiological characteristics associated with drought tolerance, including transpiration and photosynthesis rate, have been identified and provide opportunities to breed high-yielding drought-tolerant genotypes (Pimratch et al., 2008; Balota et al., 2012). RNA-sequencing (RNA-Seq), a technique for genome-wide gene expression analysis, provides a powerful alternative to facilitate the development of drought-tolerant genotypes (Li et al., 2014; Lu et al., 2014; Zhao et al., 2018). Recently, candidate genes and expression profiles in many crops, including wheat, corn, soybean, and peanut, evaluating plant response to environmental stress conditions were determined with RNA-Seq technology (Mathioni et al., 2011; Petre et al., 2012; Chen et al., 2013; Li et al., 2014; Brasileiro et al., 2015; Ruan et al., 2018; Zhao et al., 2018; Long et al., 2019). Large-scale screening of peanut had identified some drought-related candidate genes such as basic leucine zipper (*bZIP*) transcription factor genes that were observed from the wild relative of cultivated peanut, *Arachis duranensis*, when the plant was subjected to drought with 18% soil water content (Guimarães et al., 2012). Likewise, Li et al. (2014) reported 621 genes that were rapidly induced under water deficit conditions and the key drought response mechanisms in peanut function through the abscisic acid (ABA)-dependent pathway. In addition, more than 4,000 genes were identified to be associated with drought stress, in which 224 transcription factors and genes were involved in photosynthesis-antenna proteins, carbon metabolism, and the citrate cycle (Zhao et al., 2018).

As the genome sequence of the cultivated peanut cultivar Tifrunner was released (Bertioli et al., 2019), a more accurate transcriptome assembly can be obtained by mapping to the reference genome. Thus, the present study’s objective was to discover drought-induced genes by comparing drought-tolerant with drought-susceptible lines under drought stress by mapping RNA-Seq data to the cultivated peanut reference genome. The data generated in this research will provide insights into molecular mechanisms that underlie drought tolerance and provide a novel resource to further advance molecular breeding research in peanut.

## Materials and Methods

### Plant Materials and Experimental Design

The experiments were performed using four cultivated peanut genotypes, Tifrunner (susceptible), C76-16 (tolerant), 587 (tolerant), and 506 (susceptible), which were selected based on the drought study conducted in 2015 and 2016. The genotypes 587 and 506 are two recombinant inbred lines (RILs) derived from the cross “Tifrunner × C76-16,” representing the highest and lowest drought-tolerant level. The “Tifrunner × C76-16” RIL population was one of the 16 nested mapping populations (Holbrook et al., 2013). A split-plot design with randomized complete block design within was adopted in this study. All seeds were planted in a single-row (15 × 120 cm) at a rate of 10 seeds m^–1^ under rainout shelters at the United States Department of Agriculture (USDA) Agricultural Research Service National Peanut Research Laboratory in Dawson, GA, United States, to create artificial drought stress. Two rainout shelters were designated with two treatments, including full irrigation and middle-season drought, and each shelter consists of three blocks. Irrigation was provided for both treatments right after seed sowing to encourage uniform germination. The irrigated treatment (designated as “irrigated control”) received a full irrigation schedule throughout the growing season based on the evapotranspiration replacement described by Stansell et al. (1976). The drought treatment (designated as “treatment”) was fully irrigated at the beginning of the growing season until 61 days after planting. Water-deficit stress was applied at 61 days after planting by withheld water for four consecutive weeks starting at −10 kPa of soil water potential at 20 cm depth and progressively advanced to −700 kPa after 1 week of treatment and reached −1,050 to −1,200 kPa in the second week and stabilized for another 2 weeks. Specific leaf area, ^15^N and ^13^C natural abundance were determined to reflect physiological responses to drought stress based on the method described by Dang et al. (2012). Besides the water treatment, all other agronomic management practices were applied according to the University of Georgia’s best management practices for peanuts.

### RNA Extraction and Library Construction

Fully expanded leaves (second nodal) were randomly collected from each genotype at the end of the drought period in 2016. Leaf samples of each genotype were flash-frozen using liquid nitrogen and stored at −80°C until RNA extraction. Three leaflets randomly collected from each biological replication were pooled, and approximately 0.2-g pooled leaf samples were ground in liquid nitrogen for RNA extraction. Total RNA was extracted using a modified cetyltrimethylammonium bromide method (Yin et al., 2011) and purified using a Direct-Zol RNA MiniPrep Kit (Zymo Research, Irvine, CA, United States). The purity and integrity of RNA were analyzed using NanoDrop ND-1000 UV/Vis spectrophotometer (Thermo Scientific, Wilmington, DE, United States) and Agilent 2100 Bioanalyzer (Agilent, United States), respectively. A total of 24 libraries of complementary DNA (4 genotypes × 2 treatments × 3 replicates) were constructed and subsequently sequenced using an Illumina HiSeq 4000 instrument at the Beijing Genomics Institute.

### Quantitative Reverse Transcription-Polymerase Chain Reaction Validation of Differentially Expressed Genes

Quantitative reverse transcription-polymerase chain reaction (qRT-PCR) was applied to verify the transcription levels of 14 randomly selected genes. The same RNA samples in high-throughput sequencing were used for qRT-PCR. Gene-specific primers were designed by Primer Premier 3.0 software (Supplementary Table 1). Each 10-μl qRT-PCR reaction mixture contained 1 μl of 10-fold diluted first-strand complementary DNA, 0.3 μl of each primer (10 μM), and 5-μl 2 × PowerUP^TM^ SYBR^TM^ Green Master Mix (Applied Biosystems, Carlsbad, CA, United States). A Bio-Rad CFX96 real-time PCR system was used under the following conditions: 50°C for 2 min, 95°C for 2 min, followed by 40 cycles of 95°C for 15 s and 60°C for 1 min. For normalizing expression levels, yellow-leaf-specific protein 8 (NM_120912) was used as a reference gene. Non-specific products were identified by melting-curve analysis (17 genes reduced to 14 genes due to elimination of non-specific amplification). The quantification cycle value of each gene and RNA-seq results are listed in Supplementary Table 2. Relative gene expression levels were calculated using the 2^–△△*Ct*^ method (Livak and Schmittgen, 2001).

### Bioinformatics Analysis

#### Quality Control, Alignment, and Genome-Guided Assembly

The raw reads from RNA-seq were trimmed with Trimmomatic (Bolger et al., 2014). Clean reads were obtained by removing the adaptor sequences, ambiguous “N” nucleotides, and low-quality reads from the raw data. The read quality was assessed using FastQC (Andrews, 2010) before and after trimming. High-quality clean data were subjected to the downstream analyses. The RNA-seq data analysis pipeline followed the protocol described by Trapnell et al. (2012). Each sample was mapped to the reference genome by Tophat2 (Kim et al., 2013), with all the parameters setting to default. The cultivated peanut genome and the annotation file (Bertioli et al., 2019) were used as a reference for alignment. The alignment files of the 24 samples from Tophat2 were input into Cufflinks (Trapnell et al., 2012) for transcripts reconstruction.

To identify the novel transcript sequences, all the assemblies were compared with the reference annotation using Cuffcompare. Novel transcript sequences were then compared with the “Nr” database at National Center for Biotechnology Information by BLASTX to achieve gene functional annotation.

#### Identification of Differentially Expressed Genes

The expected number of fragments per kilobase of transcript sequence per millions of base pairs sequenced was used to represent the gene expression levels based on the length of the gene and reads count mapped to this gene. The differentially expressed genes (DEGs) analysis was performed using Cuffdiff [false discovery rate (FDR) < 0.05] (Trapnell et al., 2012). By comprising gene expression between “irrigated control” and “drought treatment” samples for each genotype, the DEGs were identified. The calculated *P-*value was then adjusted through FDR correction. Genes with adjusted *P-*values < 0.05 were considered as significantly differentially expressed.

#### Gene Ontology Enrichment Analysis and Kyoto Encyclopedia of Genes and Genomes Pathway Analysis

Gene ontology (GO) terms for gene models accessible in genome annotation were directly retrieved from the “GFF” file downloaded at PeanutBase^1^. GO terms for the novel transcripts were assigned using Blast2Go (Conesa et al., 2005). To combine the GO terms of the annotated genes and novel genes, the GO enrichment analysis for DEGs was performed using AgriGO^2^ (Tian et al., 2017). GO terms with FDR-adjusted *P-*value < 0.05 were considered as significantly enriched by DEGs. The enriched GO terms were subsequently visualized using REVIGO (Supek et al., 2011). To identify important pathways involved by the DEGs, the transcripts were assigned to the Kyoto Encyclopedia of Genes and Genomes (KEGG) pathways using the webserver^3^ against the *Arabidopsis thaliana, Glycine max*, *A. duranensis*, and *Arachis ipaensis* gene datasets using the bidirectional best hit method. KEGG enrichment analysis was conducted on the KOBAS 3.0 webserver^4^ (Wu et al., 2006).

## Results

We have observed progressive changes of soil water potential at 20 cm depth starting at −10 kPa advanced to −700 kPa after 1-week treatment and reached −1,050 to −1,200 kPa in the second week and stabilized for another 2 weeks. After 4 weeks of middle-season drought stress treatment, specific leaf area for these four genotypes were 34.85 ± 2.90 for C76-16, 33.41 ± 3.17 for 587, 29.40 ± 4.95 for Tifrunner, and 28.89 ± 2.97 for 506 compared with irrigated treatment at 30.31 ± 4.6 for C76-16, 32.22 ± 0.65 for 587, 31.89 ± 1.45 for Tifrunner, and 40.65 ± 0.64 for 506, indicating that these genotypes have different levels of physiological response to drought stress. There are significant differences found among the four genotypes under drought stress *vs*. non-differences under irrigation for ^15^N and ^13^C natural abundances (Wang et al., 2021 under review).

### Genome-Guided Assembly and Annotation of Novel Transcripts

To assess the global transcriptome profile of peanut leaf samples in response to drought stress, RNA-Seq analysis was performed using peanut leaves under drought treatments. RNA-seq of 24 samples of the four genotypes with three replicates under “irrigated control” or “treatment” generated a total of 1,059,869,097 pairs of 100-bp cleaned reads (197.41 Gb) with an average of 44.16 million read pairs per library representing coverage of 77.27 times. After trimming, 87.81% of the raw reads, including 930,991,527 paired reads and 77,462,493 unpaired reads, high-quality and vector-trimmed sequences were retained (Table 1). The cleaned reads were mapped to the cultivated peanut genome, and the overall mapping rate per library ranged from 62.20 to 79.30%, with an average mapping rate of 73.42% (Table 1).

**TABLE 1:** Summary of library, trimming, and alignment of reads to *A. hypogaea* genome in each library.

Genotype	Sample type	Input read pairs	Both surviving	Forward only surviving	Reverse only surviving	Dropped	Overall alignment rate (%)
Tifrunner	Control	43,170,429	37,947,881 (87.90%)	3,481,624 (8.06%)	608,537 (1.41%)	1,132,387 (2.62%)	77.60
Tifrunner	Control	48,861,831	43,973,662 (90.00%)	3,082,838 (6.31%)	754,300 (1.54%)	1,051,031 (2.15%)	73.40
Tifrunner	Control	43,839,412	39,489,936 (90.08%)	2,774,014 (6.33%)	685,366 (1.56%)	890,096 (2.03%)	76.90
587	Control	40,070,955	36,200,463 (90.34%)	2,424,462 (6.05%)	624,743 (1.56%)	821,287 (2.05%)	79.30
587	Control	44,781,655	40,323,747 (90.05%)	2,875,811 (6.42%)	656,639 (1.47%)	925,458 (2.07%)	72.90
587	Control	44,683,142	40,559,180 (90.77%)	2,486,187 (5.56%)	704,413 (1.58%)	933,362 (2.09%)	73.20
506	Control	46,447,905	41,940,433 (90.30%)	2,887,090 (6.22%)	689,080 (1.48%)	931,302 (2.01%)	64.80
506	Control	47,081,614	42,489,538 (90.25%)	2,967,321 (6.30%)	710,662 (1.51%)	914,093 (1.94%)	65.90
506	Control	48,467,014	43,354,569 (89.45%)	3,122,297 (6.44%)	839,644 (1.73%)	1,150,504 (2.37%)	75.50
C76-16	Control	44,107,057	30,307,111 (68.71%)	1,502,648 (3.41%)	9,723,368 (22.04%)	2,573,930 (5.84%)	67.90
C76-16	Control	44,738,941	39,605,524 (88.53%)	3,111,979 (6.96%)	757,947 (1.69%)	1,263,491 (2.82%)	62.20
C76-16	Control	44,941,724	40,406,587 (89.91%)	2,744,593 (6.11%)	770,588 (1.71%)	1,019,956 (2.27%)	67.70
Tifrunner	Treatment	44,640,385	39,841,817 (89.25%)	2,962,824 (6.64%)	805,756 (1.80%)	1,029,988 (2.31%)	78.70
Tifrunner	Treatment	34,479,724	30,039,097 (87.12%)	2,622,507 (7.61%)	691,319 (2.01%)	1,126,801 (37.00%)	78.50
Tifrunner	Treatment	44,888,435	39,964,474 (89.03%)	3,022,199 (6.73%)	830,020 (1.85%)	1,071,742 (2.39%)	77.40
506	Treatment	43,660,502	39,093,910 (89.54%)	2,863,022 (6.56%)	722,662 (1.66%)	980,908 (2.25%)	70.80
506	Treatment	37,280,523	32,601,953 (87.45%)	3,329,725 (8.93%)	591,735 (1.59%)	757,110 (2.03%)	78.60
506	Treatment	50,216,886	43,881,482 (87.38%)	4,466,693 (8.89%)	803,551 (1.60%)	1,065,160 (2.12%)	71.50
587	Treatment	49,500,427	43,151,072 (87.17%)	4,570,321 (9.23%)	773,842 (1.56%)	1,005,192 (2.03%)	74.40
587	Treatment	41,764,992	36,311,859 (86.94%)	3,889,085 (9.31%)	667,580 (1.60%)	896,468 (2.15%)	75
587	Treatment	44,072,172	38,394,486 (87.12%)	4,033,529 (9.15%)	702,205 (1.59%)	941,952 (2.14%)	75.10
C76-16	Treatment	47,419,319	41,051,649 (86.57%)	4,630,751 (9.77%)	720,477 (1.52%)	1,016,442 (2.14%)	74.90
C76-16	Treatment	40,858,205	35,399,986 (86.64%)	3,911,482 (9.57%)	635,605 (1.56%)	911,132 (2.23%)	72.40
C76-16	Treatment	39,895,848	34,661,111 (86.88%)	3,699,491 (9.27%)	652,797 (1.64%)	882,449 (2.21%)	77.40

Through the genome-guided assembly, a total of 73,575 genes were assembled for Tifrunner, 73,610 genes were assembled for C76-16, 73,898 genes were assembled for 587, and 73,900 genes were assembled for 506. There were 66,437 (90.30%), 66,445 (90.27%), 66,373 (89.82%), and 66,378 (89.82%) assembled genes matched to genes annotated from the cultivated peanut reference genome in Tifrunner, C76-16, 587, and 506, respectively (Table 2), resulting in 7,138, 7,165, 7,525, and 7,522 novel genes that were identified in Tifrunner, C76-16, 587, and 506, respectively.

**TABLE 2:** Summary of library and alignment of reads to *A. hypogaea* genome in each genotype.

Genotype	Total genes	Annotated	DEGs	Upregulated	Downregulated	Annotated DEGs
Tifrunner	73,575	66,437	7,780	5,310	2,470	6,410
587	73,898	66,373	13,005	7,718	5,287	10,605
506	73,900	66,378	9,767	6,052	3,715	8,065
C76-16	73,610	66,445	12,348	7,172	5,176	10,210

### Differentially Expressed Genes

The DEGs were determined between “irrigated control” and “treatment” samples of each genotype. For the drought-susceptible genotypes, there were 7,780 genes differentially expressed in Tifrunner and 9,767 differentially expressed in 506 (Table 2). Of the 7,780 DEGs in Tifrunner, the levels of gene expression of 5,310 genes were increased, and 2,470 genes were decreased. For genotype 506, 6,052 genes were upregulated, and 3,715 genes were downregulated in the drought treatment (Table 2). For the drought-tolerant genotypes, 12,348 DEGs were identified in C76-16, including 7,172 upregulated genes and 5,176 downregulated genes. In addition, a total of 13,005 DEGs were identified in 587 to be upregulated (7,718 genes) or downregulated (5,287 genes). Among the DEGs identified, 6,410, 10,210, 10,605, and 8,065 DEGs were annotated with the reference genome in Tifrunner, C76-16, 587, and 506, respectively (Table 2).

**FIGURE 1:**
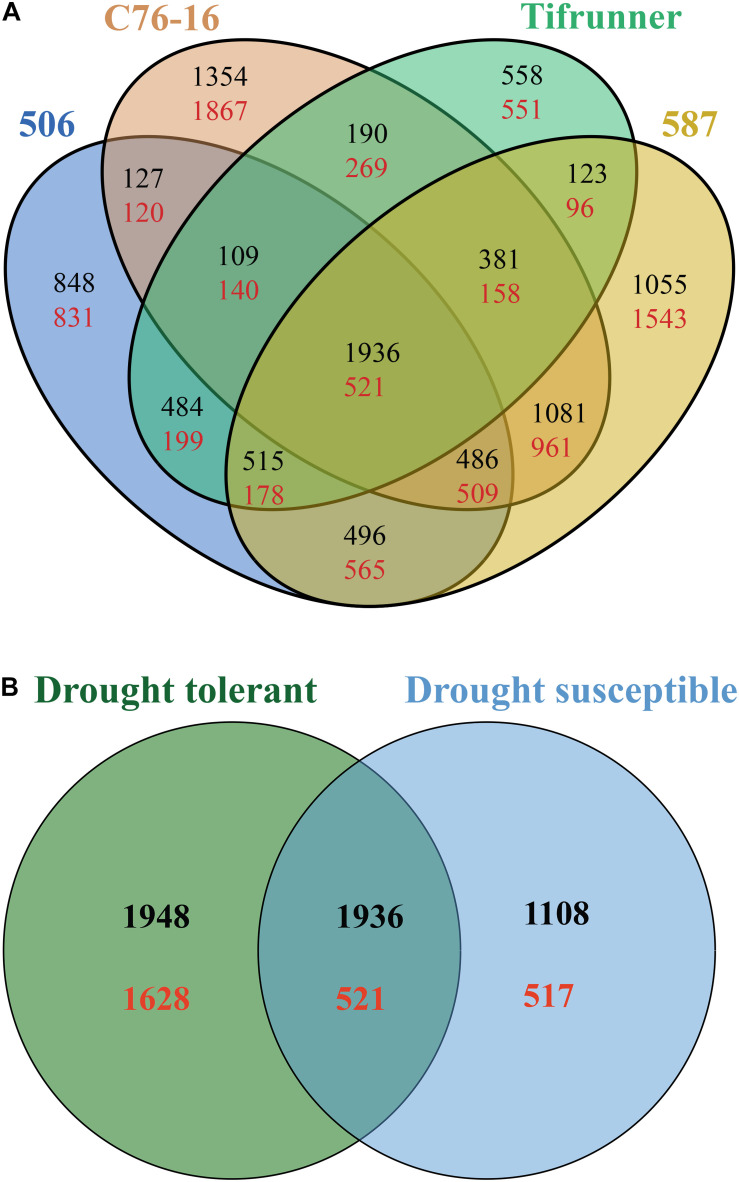
Comparison of the annotated DEGs among the four genotypes **(A)** and among the drought-susceptible genotypes, Tifrunner, and 506, and among drought-tolerant genotypes, C76-16, and 587 **(B)**. Black color numbers present upregulated genes, and red color numbers present downregulated genes.

Pairwise comparison of the DEGs from the four genotypes was performed to investigate which genes failed to respond to drought stress in drought-susceptible genotypes and in drought-tolerant genotypes (Figure 1A). A total of 5,703 DEGs, including 3,668 upregulated genes and 2,035 downregulated genes, were shared by 506 and two drought-tolerant genotypes (Figure 1A). Tifrunner shared 4,611 DEGs (3,246 upregulated and 1,365 downregulated) with the drought-tolerant genotypes (Figure 1A). Among the identified DEGs in the drought-tolerant lines, 3,860 genes were shared between 587 and C76-16, with 10,315 DEGs exclusively detected in the two drought-tolerant genotypes. Moreover, there were 2,457 DEGs identified in all four genotypes, and these genes were used in the subsequent GO and KEGG enrichment analysis.

Among the identified DEGs in the drought-tolerant lines, 6,033 genes (3,884 upregulated genes and 2,149 downregulated genes) were shared between 587 and C76-16. A total of 4,082 shared DEGs with 3,044 upregulated and 1,038 downregulated genes in drought-susceptible lines (Tifrunner and 506). A total of 2,457 DEGs were shared between drought-tolerant lines and drought-susceptible lines with 1,936 upregulated and 521 downregulated genes. In addition, 3,576 DEGs were identified in both drought-tolerant lines but were not identified in drought-susceptible lines. Also, 1,625 genes were differentially expressed in drought-susceptible lines, which were not in drought-tolerant lines (Figure 1B).

**FIGURE 2:**
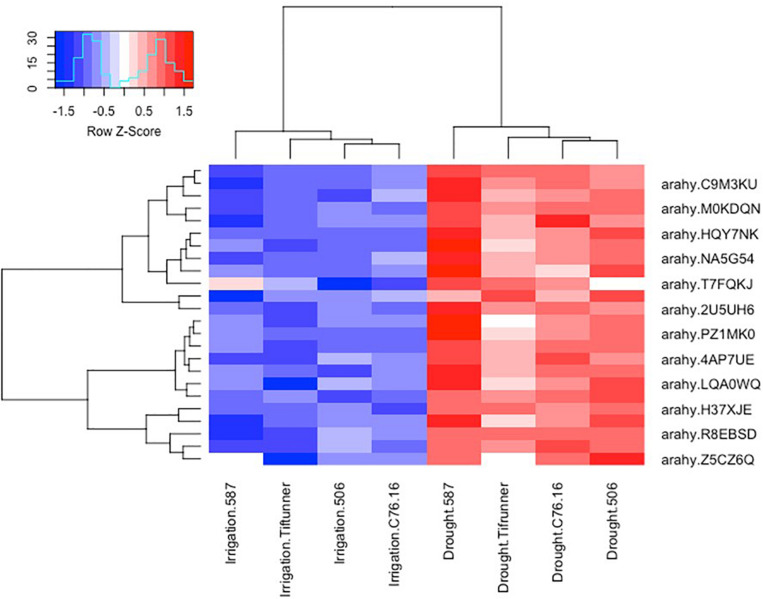
Expression profiles of the ABA-related differentially expressed genes shared by all four genotypes under irrigated and drought treatments. Log_10_ transformed FPKM values were used. “Blue” color indicates no expression or low expression level, and “red” color indicates high expression level. arahy.C9M3KU, myb transcription factor; arahy.M0KDQN, F-box family protein; arahy.HQY7NK, glucan endo-1,3-beta-glucosidase-like; arahy.NA5G54, WRKY family transcription factor; arahy.T7FQKJ, asparagine synthetase 3; arahy.2U5UH6, subtilisin-like serine protease; arahy.PZ1MK0, myb transcription factor; arahy.4AP7UE, U-box domain-containing protein 21-like; arahy.LQA0WQ, glutathione *S*-transferase family protein; arahy.H37XJE, calcium-dependent lipid-binding (CaLB domain) family protein; arahy.R8EBSD, RING-H2 finger protein 2B; IPR013083 (link is external) (zinc finger, RING/FYVE/PHD-type); arahy.Z5CZ6Q (link is external) glutathione *S*-transferase family protein.

**FIGURE 3:**
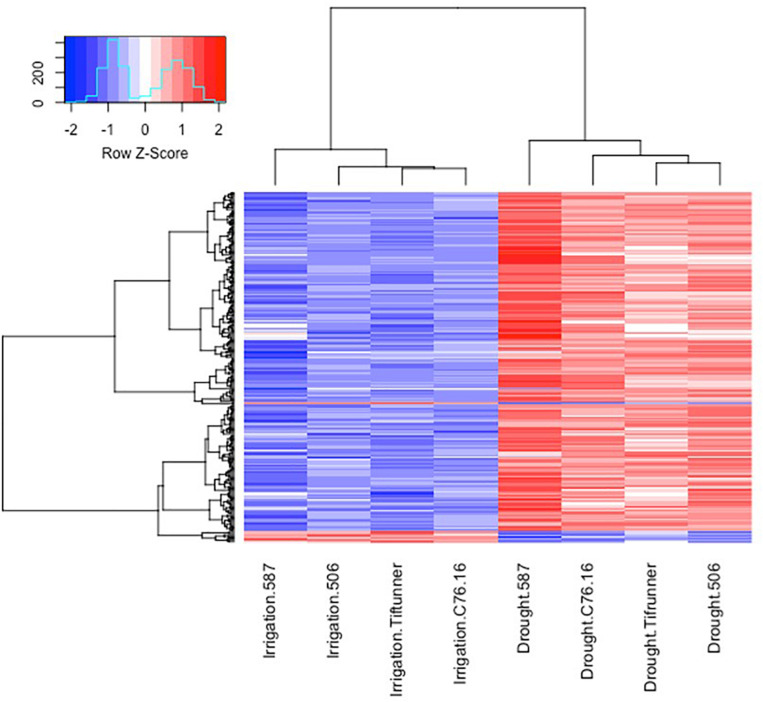
Expression profiles of the differentially expressed genes shared by all four genotypes under irrigated and drought treatments. Log_10_ transformed FPKM values were used. “Blue” color indicates no expression or low expression level, and “red” color indicates high expression level

Among the 2,457 DEGs shared by all four genotypes, a log_2_-fold change of >2 and <−2 thresholds was used to select the most significant DEGs. After filtering, only 250 genes were determined as significant DEGs resulting in 78 unique genes with their expression profiles shown in Figures 2, 3. Among these 78 genes, 76 were upregulated under drought stress, whereas 2 were downregulated.

### Gene Ontology Enrichment and Functional Classification of Differentially Expressed Genes

Gene ontology assignments were used to classify the functions of DEGs. GO enrichment analysis was performed on the 2,457 genes to identify processes and functions overrepresented in the DEGs. The 2,457 drought-responsive DEGs were assigned into 139 enriched GO terms consisting of 86 biological processes and 53 molecular functions (Table 3). In addition, a total of 74 GO terms were identified in the DEGs expressed only in drought-tolerant lines, including 55 biological processes and 19 molecular functions, mainly related to protein modification process, pollination, and metabolic process (Supplementary Table 3). These GO terms were also identified in DEGs shared in both drought-tolerant lines and drought-susceptible lines, indicating that drought-tolerant lines adjusted more genes to respond more positively to drought. We also analyzed the enrichment of DEGs expressed only in drought-susceptible lines and identified 32 GO terms (Supplementary Table 3).

**TABLE 3:** Enriched GO terms of the DEGs common in drought-tolerant genotypes and drought-susceptible genotypes.

GO term	Description	Number in input list	Number in BG/Ref	FDR
GO:0006464	Cellular protein modification process	1,137	3,209	4.10E-22
GO:0036211	Protein modification process	1,137	3,209	4.10E-22
GO:0043412	Macromolecule modification	1,178	3,355	1.50E-21
GO:0006468	Protein phosphorylation	940	2,637	2.00E-19
GO:0006793	Phosphorus metabolic process	1,229	3,587	1.20E-18
GO:0006796	Phosphate-containing compound metabolic process	1,225	3,579	1.60E-18
GO:0016310	Phosphorylation	998	2,905	1.10E-15
GO:0008037	Cell recognition	98	181	3.40E-12
GO:0009856	Pollination	98	181	3.40E-12
GO:0044706	Multi-multicellular organism process	98	181	3.40E-12
GO:0048544	Recognition of pollen	98	181	3.40E-12
GO:0009875	Pollen-pistil interaction	98	181	3.40E-12
GO:0044702	Single organism reproductive process	105	202	1.30E-11
GO:0032501	Multicellular organismal process	113	225	2.80E-11
GO:0044703	Multi-organism reproductive process	105	206	5.80E-11
GO:0007154	Cell communication	435	1,193	8.90E-11
GO:0022414	Reproductive process	109	220	1.90E-10
GO:0000003	Reproduction	109	220	1.90E-10
GO:0051704	Multi-organism process	108	218	2.20E-10
GO:0008152	Metabolic process	5,124	17,453	1.20E-08
GO:0044699	Single-organism process	2,592	8,647	1.30E-07
GO:0050789	Regulation of biological process	992	3,152	1.60E-06
GO:0065007	Biological regulation	1,051	3,355	1.70E-06
GO:0050794	Regulation of cellular process	974	3,097	2.30E-06
GO:0009765	Photosynthesis, light harvesting	23	30	4.20E-06
GO:0006470	Protein dephosphorylation	63	128	1.20E-05
GO:0010468	Regulation of gene expression	569	1,758	3.40E-05
GO:0044710	Single-organism metabolic process	1,757	5,879	4.20E-05
GO:1901565	Organonitrogen compound catabolic process	59	121	4.20E-05
GO:0051252	Regulation of RNA metabolic process	554	1,712	4.50E-05
GO:0019219	Regulation of nucleobase-containing compound metabolic process	561	1,738	5.20E-05
GO:2001141	Regulation of RNA biosynthetic process	552	1,709	5.30E-05
GO:0006355	Regulation of transcription, DNA-templated	552	1,709	5.30E-05
GO:1903506	Regulation of nucleic acid-templated transcription	552	1,709	5.30E-05
GO:0016567	Protein ubiquitination	55	112	6.50E-05
GO:0044237	Cellular metabolic process	3,180	10,932	7.10E-05
GO:0051171	Regulation of nitrogen compound metabolic process	563	1,752	7.60E-05
GO:0010556	Regulation of macromolecule biosynthetic process	557	1,734	8.20E-05
GO:2000112	Regulation of cellular macromolecule biosynthetic process	557	1,734	8.20E-05
GO:0009889	Regulation of biosynthetic process	557	1,734	8.20E-05
GO:0071704	Organic substance metabolic process	3,747	12,961	8.20E-05
GO:0031326	Regulation of cellular biosynthetic process	557	1,734	8.20E-05
GO:0044763	Single-organism cellular process	1,501	5,011	0.0001
GO:0032446	Protein modification by small protein conjugation	55	114	0.00011
GO:0044711	Single-organism biosynthetic process	446	1,366	0.00013
GO:0009987	Cellular process	3,940	13,694	0.00021
GO:0007165	Signal transduction	334	999	0.00025
GO:0023052	Signaling	334	999	0.00025
GO:0044700	Single organism signaling	334	999	0.00025
GO:0044267	Cellular protein metabolic process	1,392	4,656	0.00027
GO:0070647	Protein modification by small protein conjugation or removal	57	123	0.00029
GO:0016311	Dephosphorylation	77	180	0.00029
GO:0050896	Response to stimulus	844	2,748	0.00038
GO:0060255	Regulation of macromolecule metabolic process	579	1,834	0.00038
GO:0044238	Primary metabolic process	3,472	12,070	0.00052
GO:0006950	Response to stress	575	1,827	0.00058
GO:0019222	Regulation of metabolic process	579	1,841	0.00058
GO:0043648	Dicarboxylic acid metabolic process	48	102	0.00093
GO:0009628	Response to abiotic stimulus	49	105	0.00098
GO:0031323	Regulation of cellular metabolic process	564	1,800	0.0011
GO:0080090	Regulation of primary metabolic process	564	1,802	0.0012
GO:0006952	Defense response	342	1,056	0.003
GO:0044260	Cellular macromolecule metabolic process	2,328	8,056	0.003
GO:0055114	Oxidation-reduction process	984	3,302	0.0068
GO:0009308	Amine metabolic process	49	113	0.0094
GO:0044281	Small molecule metabolic process	595	1,948	0.011
GO:0019538	Protein metabolic process	1,629	5,612	0.013
GO:0043170	Macromolecule metabolic process	2,597	9,085	0.013
GO:0015914	Phospholipid transport	19	33	0.016
GO:0015748	Organophosphate ester transport	19	33	0.016
GO:0006022	Aminoglycan metabolic process	24	46	0.019
GO:0009266	Response to temperature stimulus	35	77	0.027
GO:0009408	Response to heat	35	77	0.027
GO:0006040	Amino sugar metabolic process	24	47	0.027
GO:0044723	Single-organism carbohydrate metabolic process	237	731	0.032
GO:0044283	Small molecule biosynthetic process	190	573	0.032
GO:0034654	Nucleobase-containing compound biosynthetic process	709	2,372	0.032
GO:1901362	Organic cyclic compound biosynthetic process	794	2,676	0.037
GO:0016053	Organic acid biosynthetic process	161	479	0.041
GO:0009066	Aspartate family amino acid metabolic process	29	62	0.043
GO:0009081	Branched-chain amino acid metabolic process	14	23	0.045
GO:0006629	Lipid metabolic process	319	1,017	0.045
GO:0009082	Branched-chain amino acid biosynthetic process	10	14	0.045
GO:0051716	Cellular response to stimulus	398	1,292	0.049
GO:0043650	Dicarboxylic acid biosynthetic process	8	10	0.049
GO:0006537	Glutamate biosynthetic process	8	10	0.049
GO:0004674	Protein serine/threonine kinase activity	722	1,849	5.30E-26
GO:0016301	Kinase activity	1,072	2,982	2.10E-23
GO:0004672	Protein kinase activity	957	2,649	7.90E-22
GO:0016773	Phosphotransferase activity, alcohol group as acceptor	1,055	2,984	1.00E-20
GO:0016740	Transferase activity	2,096	6,693	4.20E-12
GO:0016772	Transferase activity, transferring phosphorus-containing groups	1,218	3,740	1.20E-11
GO:0043565	Sequence-specific DNA binding	285	714	1.20E-11
GO:0003700	Transcription factor activity, sequence-specific DNA binding	364	1,021	1.50E-07
GO:0001071	Nucleic acid binding transcription factor activity	364	1,021	1.50E-07
GO:0003824	Catalytic activity	5,256	18,125	2.20E-06
GO:0030554	Adenyl nucleotide binding	1,753	5,820	1.10E-05
GO:0032559	Adenyl ribonucleotide binding	1,727	5,732	1.10E-05
GO:0005506	Iron ion binding	322	927	1.80E-05
GO:0016791	Phosphatase activity	121	299	5.00E-05
GO:0042578	Phosphoric ester hydrolase activity	159	418	8.10E-05
GO:0005524	ATP binding	1,447	4,811	0.0001
GO:0016597	Amino acid binding	41	76	0.0001
GO:0004721	Phosphoprotein phosphatase activity	71	157	0.00011
GO:0030246	Carbohydrate binding	143	375	0.00019
GO:0004722	Protein serine/threonine phosphatase activity	38	70	0.00019
GO:0001883	Purine nucleoside binding	1,858	6,291	0.00027
GO:0017076	Purine nucleotide binding	1,887	6,394	0.00027
GO:0032555	Purine ribonucleotide binding	1,858	6,291	0.00027
GO:0032550	Purine ribonucleoside binding	1,858	6,291	0.00027
GO:0032553	Ribonucleotide binding	1,884	6,388	0.0003
GO:0001882	Nucleoside binding	1,863	6,325	0.00042
GO:0032549	Ribonucleoside binding	1,862	6,322	0.00042
GO:0097367	Carbohydrate derivative binding	1,898	6,451	0.00042
GO:0016705	Oxidoreductase activity, acting on paired donors, with incorporation or reduction of molecular oxygen	332	1,009	0.0013
GO:0043167	Ion binding	1,600	5,433	0.0014
GO:0016491	Oxidoreductase activity	1,098	3,668	0.0018
GO:0010333	Terpene synthase activity	47	101	0.0018
GO:0035639	Purine ribonucleoside triphosphate binding	1,578	5,370	0.0022
GO:0004806	Triglyceride lipase activity	36	72	0.0024
GO:0000166	Nucleotide binding	2,207	7,659	0.0073
GO:0008889	Glycerophosphodiester phosphodiesterase activity	11	14	0.0073
GO:0030247	Polysaccharide binding	56	132	0.0073
GO:0016838	Carbon-oxygen lyase activity, acting on phosphates	48	109	0.0073
GO:1901265	Nucleoside phosphate binding	2,207	7,659	0.0073
GO:0001871	Pattern binding	56	132	0.0073
GO:0051213	Dioxygenase activity	136	385	0.012
GO:0043169	Cation binding	1,478	5,075	0.013
GO:0004012	Phospholipid-translocating ATPase activity	19	33	0.015
GO:0005548	Phospholipid transporter activity	19	33	0.015
GO:0046872	Metal ion binding	1,471	5,059	0.016
GO:0020037	Heme binding	284	883	0.017
GO:0036094	Small molecule binding	2,221	7,751	0.018
GO:0031406	Carboxylic acid binding	44	103	0.026
GO:0016725	Oxidoreductase activity, acting on CH or CH2 groups	6	6	0.028
GO:0048037	Cofactor binding	362	1,158	0.028
GO:0016165	Linoleate 13S-lipoxygenase activity	22	43	0.041
GO:0070402	NADPH binding	7	8	0.043
GO:0016639	Oxidoreductase activity, acting on the CH-NH2 group of donors, NAD or NADP as acceptor	8	10	0.048

The most significantly enriched GO term in biological processes was the cellular protein modification process, followed by the protein modification process. Several other protein modification-related processes include protein dephosphorylation, protein ubiquitination, protein modification by small protein conjugation, protein metabolic process, and protein serine/threonine kinase activity, indicating the importance of protein modification in drought response. In addition, a cluster of GO terms related to defense response, such as response to stimulus, heat, abiotic variations, and temperature, were also observed. Furthermore, many reproductive-related GO terms, which included pollination, pollen–pistil interaction, reproduction, and reproductive process, were highly enriched, indicating the effects of drought stress on plant reproduction processes. There were also GO terms related to signaling (signaling and signal transduction) and regulation (regulation of transcription, RNA biosynthetic, and RNA metabolic processes) that were enriched. Additionally, kinase-related GO terms were observed, including protein serine/threonine kinase activity, kinase activity, and protein kinase activity in molecular function (Table 3). There were also many GO terms related to the oxidation-reduction processes, such as oxygen, oxidoreductase activity, and dioxygenase activity.

### Kyoto Encyclopedia of Genes and Genomes Pathway Enrichment Analysis of Differentially Expressed Genes

**FIGURE 4:**
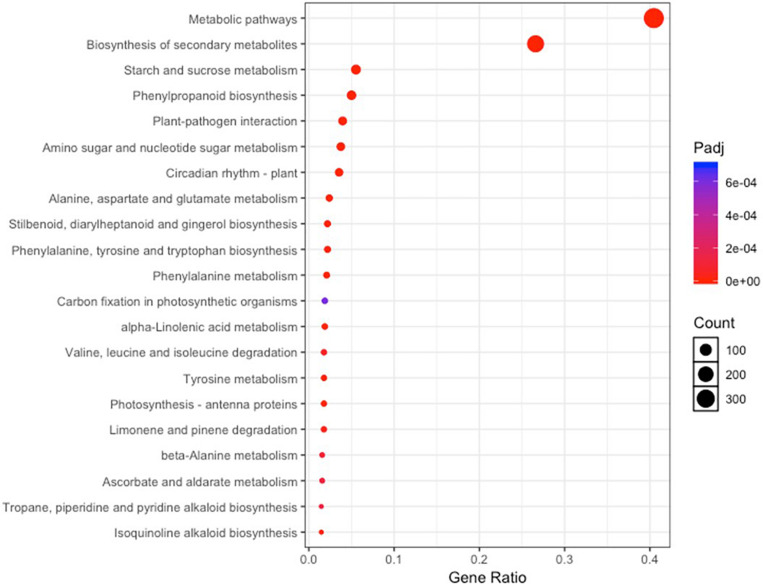
KEGG pathway enrichment analysis based on the differentially expressed genes shared by all four genotypes under irrigated and drought treatments.

A total of 741 KEGG Ontology terms were assigned to those 2,457 DEGs common in both drought-tolerant genotypes and drought-susceptible genotypes. The KEGG enrichment analysis was conducted against the *Arabidopsis thaliana* gene dataset using KOBAS 3.0 (Table 4). Forty-three pathways were identified to be significantly enriched (FDR-adjusted *P*-value < 0.05) pathways, including metabolic pathways, biosynthesis of secondary metabolites, circadian rhythm-plant, phenylpropanoid biosynthesis, starch and sucrose metabolism, etc. (Figure 4). Photosynthesis-related KEGG pathways such as photosynthesis-antenna proteins and carbon fixation in photosynthetic organisms were observed. Proline, arginine, and proline synthesis and metabolism pathways were enriched, which have been reported to be associated with drought tolerance.

**TABLE 4:** Enriched KEGG ontology terms of the DEGs common in drought-tolerant genotypes and drought-susceptible genotypes.

Term	ID	Input number	Background number	*P*-value	Corrected *P*-value
Metabolic pathways	KO01100	388	1910	7.38E-52	4.89E-50
Biosynthesis of secondary metabolites	KO01110	255	1076	4.02E-43	2.21E-41
Circadian rhythm-plant	KO04712	34	36	9.43E-20	2.70E-18
Phenylpropanoid biosynthesis	KO00940	48	157	3.10E-12	5.48E-11
Starch and sucrose metabolism	KO00500	53	202	3.16E-11	4.95E-10
Photosynthesis - antenna proteins	KO00196	17	22	1.22E-09	1.52E-08
Alanine, aspartate and glutamate metabolism	KO00250	23	48	1.77E-09	2.14E-08
Stilbenoid, diarylheptanoid, and gingerol biosynthesis	KO00945	21	46	1.73E-08	1.93E-07
Phenylalanine metabolism	KO00360	20	42	2.17E-08	2.40E-07
Amino sugar and nucleotide sugar metabolism	KO00520	36	135	3.06E-08	3.35E-07
Alpha-linolenic acid metabolism	KO00592	18	36	6.17E-08	6.41E-07
Isoquinoline alkaloid biosynthesis	KO00950	14	23	3.01E-07	2.87E-06
Phenylalanine, tyrosine and tryptophan biosynthesis	KO00400	21	57	3.17E-07	3.02E-06
Plant–pathogen interaction	KO04626	38	167	4.22E-07	3.94E-06
Tyrosine metabolism	KO00350	17	40	8.64E-07	7.55E-06
Limonene and pinene degradation	KO00903	17	44	2.46E-06	1.99E-05
Valine, leucine and isoleucine degradation	KO00280	17	48	6.36E-06	4.66E-05
Beta-alanine metabolism	KO00410	15	40	1.27E-05	8.98E-05
Ascorbate and aldarate metabolism	KO00053	15	41	1.61E-05	0.00011201
Tropane, piperidine, and pyridine alkaloid biosynthesis	KO00960	14	36	1.77E-05	0.00012194
Carbon fixation in photosynthetic organisms	KO00710	18	69	0.000104	0.00060361
Glucosinolate biosynthesis	KO00966	9	19	0.000177	0.00096339
Glutathione metabolism	KO00480	20	93	0.000408	0.00201203
Terpenoid backbone biosynthesis	KO00900	15	58	0.000412	0.00203127
Diterpenoid biosynthesis	KO00904	9	22	0.000417	0.00204405
Arginine and proline metabolism	KO00330	14	53	0.000531	0.00252985
Base excision repair	KO03410	12	43	0.000869	0.00387834
AGE-RAGE signaling pathway in diabetic complications	KO04933	8	20	0.000979	0.00428699
Biosynthesis of amino acids	KO01230	38	255	0.001263	0.00538193
Pentose and glucuronate interconversions	KO00040	17	81	0.001351	0.00572059
Cysteine and methionine metabolism	KO00270	21	112	0.001387	0.00584628
Nucleotide excision repair	KO03420	15	69	0.001878	0.0077515
Ubiquinone and other terpenoid-quinone biosynthesis	KO00130	10	35	0.001983	0.00814361
Inositol phosphate metabolism	KO00562	14	68	0.004012	0.01497196
Thiamine metabolism	KO00730	5	11	0.005897	0.02069711
Monoterpenoid biosynthesis	KO00902	4	7	0.00763	0.02585832
Pyruvate metabolism	KO00620	15	85	0.010056	0.03283136
Porphyrin and chlorophyll metabolism	KO00860	10	48	0.012979	0.04046835
Glycine, serine and threonine metabolism	KO00260	13	72	0.013615	0.04225667
Pyrimidine metabolism	KO00240	18	116	0.015576	0.04708738
Fatty acid elongation	KO00062	8	35	0.01621	0.04871628
DNA replication	KO03030	10	50	0.016307	0.04893789
2-Oxocarboxylic acid metabolism	KO01210	13	74	0.01635	0.04899404

### Validation of RNA Sequencing Using Quantitative Reverse Transcription-Polymerase Chain Reaction

To validate the gene expression levels of the DEGs, 14 DEGs shared by all four genotypes under irrigated and drought treatments were selected for validation using qRT-PCR (Supplementary Table 2). All randomly selected 14 DEGs were confirmed to be upregulated in all four genotypes (Supplementary Table 2). Among these genes, three genes (“*Arahy.JL3DG7*,” “*Arahy.Z1CJI9*,” and “*Arahy.UKNP9M*”) were confirmed to be upregulated in all genotypes but significantly less than the expression level determined by RNA-seq. The correlation coefficient (R) between log_2_ (fold change) from RNAseq and ΔΔCq is 0.87, indicating that overall, the results of qRT-PCR agreed well with most findings from RNA-seq analysis.

## Discussion

This research aims to define potential drought tolerance mechanism(s) by defining biochemical and molecular processes between drought-tolerant peanut *versus* drought-susceptible lines. A progressive drought treatment approach, in contrast to intermittent drought, was chosen to observe a linear relationship between plant response and increasing levels of water deficit. In contrast to the survival mechanism that plants may face in severe drought and high-heat environments, the peanuts grown in the many production areas around the world look to minimize yield loss or even to increase yield under increasing drought incidence. The selection of middle season drought was chosen to observe yield differences, and relatively short drought treatment (4 weeks) provides a fine separation of peanut response comparing drought-tolerant with drought-susceptible genotypes.

Transcriptome data are valuable resources for discovering gene expression levels, characterizing new alleles, and developing molecular markers associated with drought responses by investigating plants under abiotic or biotic stress. However, studies focusing on the transcriptome of peanuts under drought stress are limited. Li et al. (2014) demonstrated 47,842 unigenes with 621 induced DEGs (≥1.5 fold change compared with control) in the seedlings of peanut (*Arachis hypogaea* L.) cultivar Yueyou7 in South China under water deficit condition, and 22 putative transcription factor (TF) genes were reported as drought-responsive. They concluded that the main drought response mechanism in peanut function was through the ABA-dependent pathway. RNA-Seq analyses on two wild relatives of cultivated peanut under drought conditions, *Arachis stenosperma* (7,722 contigs) and *A. duranensis* (12,792 contigs), classified TF transcripts into 25 and 20 families, respectively (Guimarães et al., 2012). A more recent study assembled 51,554 genes in cultivated peanut root samples under drought conditions, where 4,648 DEGs were identified by comparing the irrigation with drought treatment (Zhao et al., 2018). In contrast, the present study analyzed the transcriptome of four peanut lines by mapping the sequenced library to the cultivated peanut reference genome and thus provided a more thorough dataset showing gene regulations under drought stress. We reported 73,575, 73,898, 73,900, and 73,610 genes for Tifrunner, 587, 506, and C76-16 with 7,780, 13,005, 9,767, and 12,348 DEGs in each genotype, respectively.

The majority of the DEGs were involved in secondary metabolite biosynthesis, photosynthesis, and response to heat and abiotic stimulus. This is supported by several studies reporting plant stress resistance systems (Farooq et al., 2009; Zhao et al., 2018). Some of the DEGs in response to drought stress were involved in 11 enriched KEGG pathways (carbon fixation in photosynthetic organisms; starch and sucrose metabolism; photosynthesis-antenna proteins; porphyrin and chlorophyll metabolism; cysteine and methionine metabolism; circadian rhythm-plant; pyruvate metabolism, amino sugar, and nucleotide sugar metabolism, glycine, serine, and threonine metabolism; phenylpropanoid biosynthesis; and phenylalanine metabolism), which is consistent with the results demonstrated in a recent study on peanut (Zhao et al., 2018). DEGs enriched in carbon metabolism pathway, starch and sucrose metabolism pathway, and photosynthesis-antenna proteins suggesting plant photosynthesis were affected due to decreasing carbon dioxide assimilation rate under mid-season drought stress (Farooq et al., 2009). Similarly, several genes involved in ascorbate and aldarate metabolism, carbon fixation, and photosynthesis were also reported to drought stimuli in other plants, including *Boehmeria nivea* and *Chrysanthemum morifolium* (Liu et al., 2013; Xu et al., 2013). However, our data indicated that ribosome and plant hormone signal transduction were not enriched in peanut in response to mid-season drought, suggesting plant host, growth stages, sampling dates, and treatments might play roles in the demonstrated variability.

Drought stress significantly affected the transcripts of some key genes related to secondary metabolism. For example, protein ubiquitination is demonstrated to regulate plant drought stress response (Liu et al., 2013; Zhang et al., 2013). Our GO enrichment analysis indicated peanut ubiquitin-related genes were highly enriched in both drought-tolerant and drought-susceptible genotypes. Fifty DEGs were identified to correlate with protein ubiquitination. For example, *Arahy.4AP7UE* played a role in protein ubiquitination that may negatively regulate ABA and drought response. In support of our data, the ubiquitin-related gene *AhUBC2* was shown to enhance drought tolerance by regulating the expression of a stress-responsive gene (Wan et al., 2011).

Among the enriched KEGG and GO, many signaling-related GO terms were enriched indicating the potential importance of related pathways in peanut plants under drought stress. Interestingly, many ABA pathways related to DEGs were significantly induced by drought stress, including *Arahy.UN6GTT*, *Arahy.RRZ6LI*, *Arahy.KS1HEQ*, *Arahy.H5H05M*, and *Arahy.KLG2UC*. Plant hormones play significant roles in maintaining plants alive such as growth, under environmental stress, and senescence (Davies, 2013). ABA, as an important plant hormone being produced in the roots in response to drought, was widely studied for its role in regulating guard cell movement to close stomata (Li et al., 2014). ABA functions in plants under drought by regulating the development of reproductive tissues through massive transcriptional reprogramming events under long-term drought stress, which further reduces the plant growth and crop yield (Degenkolbe et al., 2009; Sreenivasulu et al., 2012). The previous study in peanut identified 279 DEGs that were significantly overlap in expression between the water deficit only and water deficit + ABA treatment groups, indicating the significant role of ABA in signaling under drought (Li et al., 2014). Furthermore, combining the reproduction-related pathway enriched in this study, we speculated that the induced ABA-related DEGs might further affect the reproduction process of peanuts under drought stress due to the 4-week drought period. This confirms the previous finding that ABA regulates the development of reproductive tissues under long-term drought (Degenkolbe et al., 2009; Sreenivasulu et al., 2012). Besides the ABA pathway-related genes, we also found a range of ethylene-related and auxin signaling pathway-related genes, and these genes can differentially express in the peanut under drought-stress conditions.

Late embryogenesis abundant (LEA) proteins are mainly low molecular weight (10–30 kDa) proteins, which are involved in defending higher plants from damage triggered by abiotic stresses, especially drought (dehydration). The present study identified two LEA genes (*arachy. RD0T5B* and *arachy. XWYH2Z*) that are shared by all four genotypes, five LEA genes (*arachy.3IB3IU*, *arachy.Q07BGG*, *arachy.R9W6MW*, *arachy.B0SKQG*, and *arachy.FY9BZZ*) shared uniquely in drought-tolerant genotypes, and only one LEA gene (*arachy.*P4KHGY) shared in drought-susceptible genotypes (Table 5). In our study, more LEA genes were upregulated in drought-tolerant genotypes than in drought-susceptible genotypes. This indicated the essential role of LEA proteins in peanuts under drought stress, especially in drought-tolerant genotypes. Accumulation of LEA proteins has also been found to occur in peanut roots when peanut plants under drought stress (Zhao et al., 2018). Regarding the many peanut LEA gene subfamilies, the precise functions are still enigmatic, and further research should be performed to elucidate the possible roles of these genes in peanut stress tolerance.

**TABLE 5:** Drought responsive genes, transcription factors family, and hormones may be involved in peanut tolerance to dehydration stress.

	Drought responsive genes					TF							Hormone			
			
	LEA	HSP	CHS	proline	CYP	bZip	bHLH	MYB	ARF	WRKY	NAC	ERF	JAZ	Gibberellin	Auxin	F-box
All four	2	2	4	3	7	2	4	5	1	3	0	1	7	0	0	5
C76-16 + 587	5	4	8	0	8	0	2	9	0	13	5	7	0	1	4	11
Tifrunner + 506	1	3	1	0	4	0	3	1	0	0	1	0	0	1	0	1

*LEA, late embryogenesis abundant; HSP, heat shock protein; CHS, chalcone synthase; CYP, cyclophilins; TF, transcription factor; bZip, basic leucine zipper; bHLH, basic helix-loop-helix; MYB, myeloblastosis; ARF, auxin response factor; NAC, *N*-acetyl-cysteine; EFT, ETS domain-containing transcription factor; JAZ, jasmonate ZIM domains.*

Transcription factors (sequence-specific DNA-binding factors) are proteins that bind to specific DNA sequences, thus manipulating the RNA transcription rate for genes (Latchman, 1997). TFs may perform their functions alone or with other proteins in a complex *via* promoting as an activator or blocking as a repressor the recruitment of RNA polymerase to specific genes. In legumes, different TF subfamilies might show different regulation under stress (Udvardi et al., 2007). In our study, many TFs families have been identified, and many of them have been reported to be involved in the plant drought-tolerance system (Table 5). In the present study, most of the TFs were enriched in MYB, WRKY, and ERF. In the present study, five genes from the MYB family were highly induced under drought stress in all four genotypes. In addition, nine MYB TFs were highly induced, particularly in the two drought-tolerant genotypes, and only one MYB TF was induced only in the two drought-susceptible genotypes. This indicated the significance of the MYB family in drought stress, especially in drought-tolerant genotypes. The MYB family has been documented to act through the ABA signaling cascade to regulate stomatal movement and as a result of water loss regulation in *Arabidopsis* and rice (Yanhui et al., 2006; Dai et al., 2007).

The present study demonstrated that mid-season drought alters the transcriptome profile in four peanut genotypes with varying drought-tolerant levels. Thousands of novel genes of cultivated peanuts were identified and annotated. The DEGs involved in circadian rhythm-plant, phenylpropanoid biosynthesis, starch and sucrose metabolism, photosynthesis-antenna proteins, etc., were enriched. In addition, the ABA-related pathway was considered as one of the most important mechanisms underlying drought tolerance in peanuts. This study provided insights into putative peanut response against drought stress.

Genetic variability between drought-tolerant and drought-susceptible genotypes evaluated is extremely low, suggesting that biochemical and molecular differences in drought treatment may be qualitative and subtle. The general conclusion is that the peanut drought response in this study has many similarities with other drought studies but with additive novel observations. A higher number of DEGs were observed in drought-tolerant compared with drought-susceptible lines, and there were more upregulated genes than downregulated genes in both (Supplementary Table 3). Comparing DEGs, 3,576 DEGs were observed only in drought-tolerant lines, and 1,625 DEGs were specific to drought-susceptible lines. GO terms were defined for these genes showing 74 for drought-tolerant lines highly enriched for cellular processes, protein modification, and gene-regulation and 32 for drought-susceptible lines enriched for catalytic activity, ion binding, and carbon/oxygen-binding activity. These gene activities highlight the subtleties of gene regulation demonstrated in our previous work showing drought-regulated gene expression evaluating a subset of transcription factors (Dang et al., 2012). In summary, these results showed that the mechanism underlying drought tolerance in peanuts involves a complex network of multiple hormones and a variety of molecular responses. However, the underlying regulatory mechanisms still need to be further studied. Therefore, studying plant hormone signaling pathways will be crucial in understanding the regulatory mechanism in peanut drought tolerance.

## Data Availability Statement

The datasets presented in this study can be found in online repositories. The names of the repository/repositories and accession number(s) can be found below: https://www.ncbi.nlm. nih.gov/BioSample, accessions: SAMN17150141–SAMN17150164.

## Author Contributions

CC is a director of the project. He supervised and coordinated the experiments and revised the manuscript. XW and XY conducted the experiments and wrote draft of manuscript. YF supervised RT-PCR experiments in her lab. PD performed rainout shelter drought treatment, collected leaf samples, and revised the manuscript. WW conducted data analysis for reversion. RG, JC, YC, PO-A, and CH revised the manuscript. All authors contributed to the manuscript and approved the final version of the manuscript to be published.

## Conflict of Interest

The authors declare that the research was conducted in the absence of any commercial or financial relationships that could be construed as a potential conflict of interest.

## Footnotes

1 http://peanutbase.org

2 http://systemsbiology.cau.edu.cn/agriGOv2/

3 http://www.genome.jp/kaas-bin/kaas_main

4 http://kobas.cbi.pku.edu.cn/index.php

## Supplementary Material

Supplementary Table 1 qPCR primer design.

Supplementary Table 2 Quantification calculation and comparison with RNAseq.

Supplementary Table 3 Supplemental table GO of DEGs expressed in drought tolerant genotypes and susceptible genotypes.
